# Surgical resection for esophageal adenosquamous carcinoma: an analysis of 56 cases

**DOI:** 10.1186/s12957-022-02607-0

**Published:** 2022-05-04

**Authors:** Shao-bin Chen, Di-tian Liu, Yu-ping Chen

**Affiliations:** Department of Thoracic Surgery, Cancer Hospital of Shantou University Medical College, 7 Raoping Road, Shantou, 515000 Guangdong Province China

**Keywords:** Esophagus, Adenosquamous carcinoma, Surgery, Treatment, Prognosis

## Abstract

**Background:**

Esophageal adenosquamous carcinoma (EASC) is a rare disease. The biological behavior and treatment of this malignancy are not well studied.

**Methods:**

Data from 56 patients with EASC who underwent esophagectomy were retrospectively analyzed and compared with 5028 patients with esophageal squamous cell carcinoma (ESCC). The impact of clinicopathological factors on the survival of patients with EASC was analyzed. The survival differences between patients with EASC and ESCC were also compared.

**Results:**

There were 43 males and 13 females with a mean age of 59.7 ± 1.3 years (range, 39–79 years). Only 1 of the 43 patients who received preoperative esophagoscopic biopsy was diagnosed with EASC. The median survival time for patients with EASC was 32.0 months, and the 1-, 3-, and 5-year overall survival rates were 78.3%, 46.1%, and 29.6%, respectively. Resection margin, pN category, and adjuvant chemotherapy were found to be independent predictors. After 1:1 propensity score matching, the 5-year overall survival rate of 29.6% for patients with EASC was similar to that of 42.5% for patients with ESCC (*P* = 0.179).

**Conclusions:**

EASC is a rare disease and is easily misdiagnosed by esophagoscopic biopsy. The prognosis of EASC was similar to that of ESCC. Postoperative adjuvant chemotherapy may improve the survival of patients with EASC after esophagectomy.

## Introduction

Esophageal adenosquamous carcinoma (EASC) is a rare disease that contains both squamous cell carcinoma and adenocarcinoma components with a well-defined border [1, 2]. According to a large series, EASC accounts for approximately 1% of all esophageal carcinomas [3–6]. Due to the low incidence, the biological behavior and treatment of this rare malignancy are not well established.

In a previous study [7], we reported a series of 37 patients with EASC, which was one of the largest series to date, and found that the prognosis of EASC patients was significantly poorer than that of well- or moderately differentiated esophageal squamous cell carcinoma (ESCC) patients but was comparable to that of poorly differentiated ESCC patients. However, the patient number was still too small to conduct subgroup analyses, especially in the survival analysis. We think it is necessary to perform further research to give us a better understanding of this rare disease. In this study, we reviewed data from 56 patients with EASC and aimed to investigate its biological behavior and treatment. We further used the propensity score matching (PSM) analysis to match the baseline between patients with EASC and ESCC to compare their prognosis.

## Patients and methods

A total of 5881 patients with esophageal cancer underwent esophagectomy in the Department of Thoracic Surgery, Shantou University Medical College Cancer Hospital between January 1995 and December 2019. Fifty-six patients (0.95%, 56/5881) were histopathologically diagnosed with EASC and enrolled in this study. All patients provided informed consent. This study was approved by an independent ethics committee at our hospital.

### Data collection

All clinicopathological data and laboratory data were retrospectively investigated. All specimens were re-examined by an expert pathologist (Dr. Xiao-long Wei). The histopathologic features of EASC was detail described in our previous study [7]. In briefly, the histological definition of EASC was based on the Japanese Esophageal Society classification, which required at least 20% of either the squamous cell carcinoma and adenocarcinoma components. Esophageal mucoepidermoid carcinomas was excluded from this study. Tumors were staged according to the 8th edition of the AJCC TNM staging system.

### Treatment

A left thoracotomy was routinely conducted for the patients who underwent esophagectomy before 2010, and a right thoracotomy was conducted for most of the patients after 2011. For lymphadenectomy, the paraesophageal, subcarinal, supradiaphragmatic, paracardial, lesser curvature, and left gastric lymph nodes were routinely dissected for all patients. The lymph nodes around the left and right recurrent nerves and the common hepatic lymph nodes were also resected for patients who underwent a right thoracotomy.

None of these patients received preoperative neoadjuvant therapy. Postoperative adjuvant therapy was administered to 19 patients, including 11 patients who received adjuvant radiotherapy, 5 patients who received adjuvant chemotherapy, and 3 patients who received adjuvant chemoradiotherapy.

### Statistical analysis

Statistical analysis was performed using SPSS 26.0 software (IBM, Armonk, New York, USA). Categorical variables were compared by Pearson’s chi-square test or Fisher’s exact test. Overall survival (OS) was calculated by the Kaplan–Meier method, and the log-rank test was used to assess the survival differences. Multivariate Cox regression analysis enrolled all factors with *P* < 0.2 in univariate analysis to determine the independent prognostic factors. PSM was conducted with the 1:1 nearest neighbor matching method. The covariates included sex, age, tumor location, tumor length, thoracotomy, resection margin, pT category, pN category, adjuvant radiotherapy, and adjuvant chemotherapy. *P* < 0.05 indicated statistical significance.

## Results

### Clinicopathological features of patients with EASC

The study cohort included 43 males and 13 females, and the mean age was 59.7 ± 1.3 years (range, 39–79 years). Most of the tumors (66.1%, 37/56) were located on the middle third of the thoracic esophagus, and the median length was 5.0 cm (range, 2.0–10.0 cm). Of the 43 patients who underwent preoperative esophagoscopic biopsy, only 1 patient was diagnosed with EASC, while the other forty-two patients were misdiagnosed with ESCC.

Based on the 8th edition of the AJCC TNM staging system, there was 1 case of pT1 disease, 8 cases of pT2 disease, 40 cases of pT3 disease, and 7 cases of pT4 disease. A total of 1205 lymph nodes were resected, while 118 nodes were pathologically diagnosed as metastatic. The mean number of resected lymph nodes was 21.5 ± 2.2, with a median number of 17 (range, 3–89). There were 23 cases of pN0 disease, 18 cases of pN1 disease, 10 cases of pN2 disease, and 5 cases of pN3 disease. A radical resection (complete tumor resection) was achieved in 49 patients and palliative resection (microscopically positive margins or gross positive residual margins) was performed in 7 patients.

Four patients suffered major postoperative complications, including 2 cases of pneumonia and 2 cases of esophagogastric anastomotic leaks. No patient died during treatment in the hospital.

### Comparison of clinicopathological features between patients with EASC and ESCC

Of the 5881 patients with esophageal cancer who underwent esophagectomy in our hospital between January 1995 and December 2019, 5558 patients were histopathologically diagnosed with ESCC. We excluded 477 patients who received neoadjuvant therapy (including 256 patients who received neoadjuvant chemoradiotherapy, 191 patients who received neoadjuvant radiotherapy, and 30 patients who received neoadjuvant chemotherapy), and 53 patients lacked any follow-up data. Therefore, data from a cohort of 5028 patients with ESCC were analyzed.

The clinicopathological features of the patients with EASC and ESCC are shown in Table 1. No significant differences in clinicopathological features were seen between patients with EASC and ESCC.

**Table 1:** Clinicopathological features between patients with EASC and ESCC in the original cohort and matched cohort

Variable	Original cohort		*P* value	Matched cohort		*P* value
	EASC (*n* = 56)	ESCC (*n* = 5028)		EASC (*n* = 58)	ESCC (*n* = 58)	
Sex			0.660			0.820
Male	43 (76.8%)	3731 (74.2%)		43 (76.8%)	44 (78.6%)	
Female	13 (23.2%)	1297 (25.8%)		13 (23.2%)	12 (21.4%)	
Age (year)			0.055			0.705
≤ 60	27(48.2%)	3058 (60.8%)		27(48.2%)	29(51.8%)	
> 60	29(51.8%)	1970 (39.2%)		29(51.8%)	27 (48.2%)	
Tumor location			0.737			0.965
Upper third	9 (16.1%)	655 (13.0%)		9 (16.1%)	8 (14.3%)	
Middle third	37 (66.1%)	3544 (70.5%)		37 (66.1%)	38 (67.9%)	
Lower third	10 (17.9%)	829 (16.5%)		10 (17.9%)	10(17.9%)	
Tumor length			0.081			1.000
≤ 4 cm	26 (46.4%)	1771 (35.2%)		26 (46.4%)	26 (46.4%)	
> 4 cm	30 (53.6%)	3257 (64.8%)		30 (53.6%)	30 (53.6%)	
Thoracotomy			0.183			0.841
Left thoracotomy	37 (66.1%)	3758 (74.7%)		37 (66.1%)	38(67.9%)	
Right thoracotomy	19 (33.9%)	1270 (25.3%)		19 (33.9%)	18 (32.1%)	
Resection margin			0.309			0.768
Radical	49 (87.5%)	4593 (91.3%)		49 (87.5%)	50 (89.3%)	
Palliative	7 (12.5%)	435 (8.7%)		7 (12.5%)	6 (10.7%)	
pT category			0.099			0.589
pT1-T2	9 (16.1%)	1295 (25.8%)		9 (16.1%)	7 (12.5%)	
pT3-T4	47 (83.9%)	3733 (74.2%)		47 (83.9%)	49 (87.5%)	
pN category			0.251			1.000
pN0	24 (42.9%)	2543 (50.6%)		24 (42.9%)	24 (42.9%)	
pN1-N3	32 (57.1%)	2485 (49.4%)		32 (57.1%)	32 (57.1%)	
Ajuvant radiotherapy			0.173			0.825
Yes	14 (30.4%)	1140 (22.7%)		14 (25.0%)	13 23.2%)	
No	42 (69.6%)	3888 (77.3%)		42 (75.0%)	43 (76.8%)	
Adjuvant chemotherapy			0.583			0.781
Yes	8 (14.3%)	598 (11.9%)		8 (14.3%)	7 (12.5%)	
No	48 (85.7%)	4430 (88.1%)		48 (85.7%)	49 (87.5%)	

*EASC* esophageal adenosquamous carcinoma, *ESCC* esophageal squamous cell carcinoma

### Prognosis and survival analysis for patients with EASC

Follow-up was continued to December 2020. The median survival time (MST) for 56 patients with EASC was 32.0 months (95% confidence interval [CI] 17.1-46.9), and the 1-, 3-, and 5-year OS rates were 78.3%, 46.1%, and 29.6%, respectively. In the univariate analysis, only tumor length was found to be significantly correlated with survival (*P* = 0.036, Table 2). Multivariate analysis enrolled all factors with *P* < 0.2 in univariate analysis, including tumor length, thoracotomy, resection margin, pN category, and adjuvant chemotherapy (Table 3). Resection margin, pN category, and adjuvant chemotherapy were found to be independent predictors, while tumor length and thoracotomy were not independent prognostic factors.

**Table 2:** Univariate analysis in regard to overall survival according to clinicopathological features for 56 patients with EASC

Variable	No. of patients	1-year OS (%)	3-year OS (%)	5-year OS (%)	*P* value
Sex					0.282
Male	43	71.8	42.3	21.9	
Female	13	84.6	59.2	29.2	
Age (year)					0.271
≤ 60	27	77.6	49.7	36.1	
> 60	29	71.8	42.8	22.8	
Tumor location					0.918
Upper third	9	100.0	62.5	31.3	
Middle third	37	70.3	45.4	30.3	
Lower third	10	70.0	35.0	-	
Tumor length					**0.036**
≤ 4 cm	26	84.6	56.1	41.6	
> 4 cm	30	65.5	36.4	18.2	
Thoracotomy					0.102
Left thoracotomy	37	67.6	35.1	26.4	
Right thoracotomy	19	88.2	70.1	31.5	
Resection margin					0.091
Radical	49	79.2	48.6	32.0	
Palliative	7	42.9	28.6	14.3	
pT category					0.434
pT1-T2	9	87.5	54.7	18.2	
pT3-T4	47	72.2	43.7	30.7	
pN category					0.082
pN0	24	83.3	54.2	39.4	
pN1-N3	32	67.7	40.1	21.4	
Adjuvant radiotherapy					0.667
Yes	14	78.6	50.0	28.6	
No	42	73.2	44.8	30.6	
Adjuvant chemotherapy					0.153
Yes	8	87.5	87.5	58.3	
No	48	72.3	38.9	25.0	

*OS* overall survival

**Table 3:** Multivariate analysis in regard to overall survival for 56 patients with EASC

Prognostic factor	Hazard Ratio	95%CI	*P* value
Tumor length	1.424	0.713–2.844	0.316
Thoracotomy	0.562	0.241–1.311	0.183
Resection margin	3.718	1.390–9.947	**0.009**
pN category	2.477	1.176–5.218	**0.017**
Adjuvant chemotherapy	4.197	1.153–15.280	**0.011**

*CI* confidence interval

### Comparison of survival between patients with EASC and ESCC

**Fig. 1:**
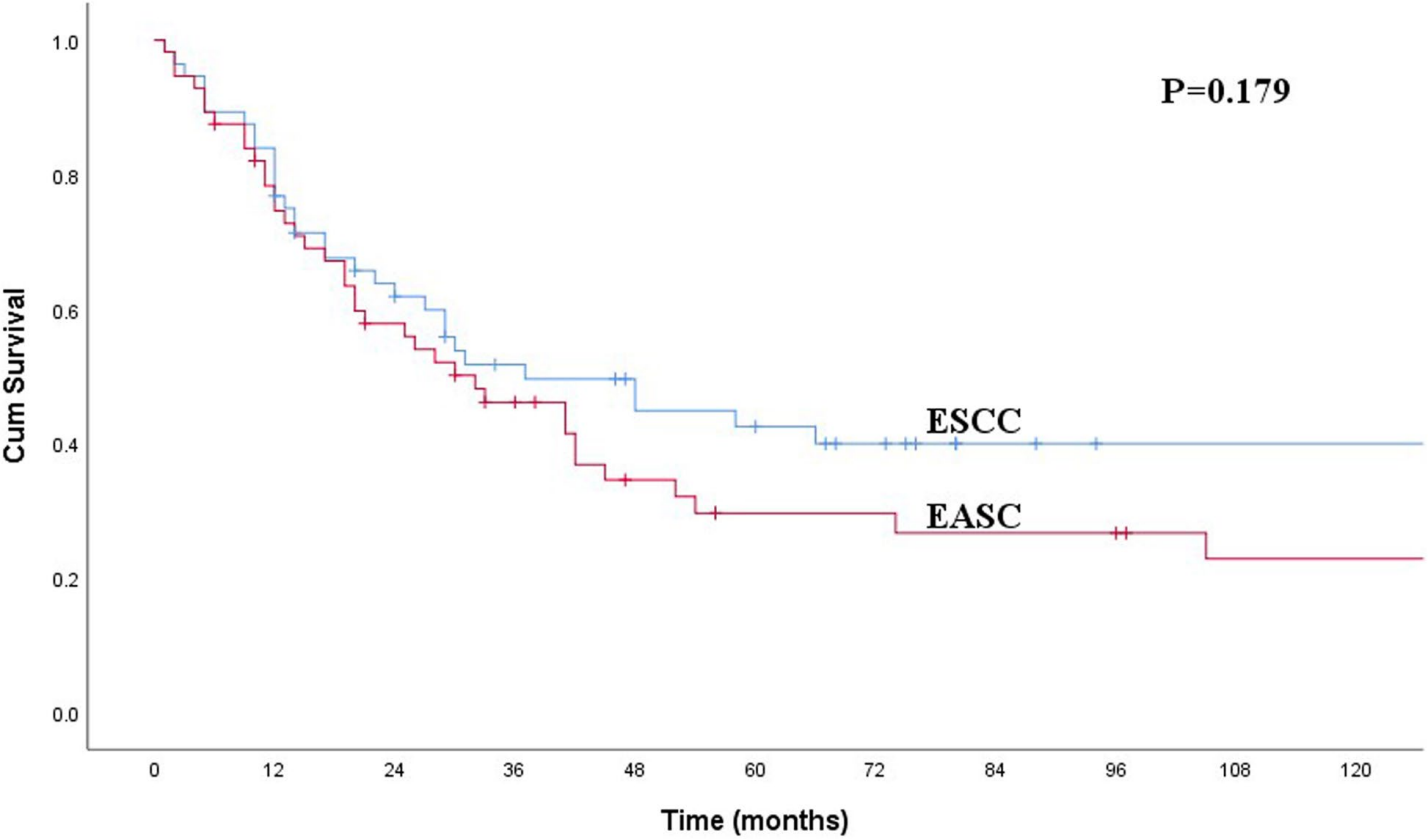
Kaplan-Meier curves for overall survival between patients with EASC and ESCC. The difference was not significant (*P* = 0.179)

In order to balance the baseline clinicopathological characteristics of patients with EASC and ESCC, we used PSM analysis to match the characteristics before survival analysis. After 1:1 propensity score matching, the clinicopathological features between patients with EASC and ESCC were balanced (Table 1). The 5-year overall survival rate was 42.5% for patients with ESCC, which was similar to that of 29.6% for patients with EASC (*P* = 0.179, Fig. 1).

## Discussion

EASC is a very rare disease and accounts for approximately 1% of all cases of primary esophageal cancer [1–6]. The biological behavior and treatment of EASC have not been well studied to date. Most of the previous studies on the disease were case reports [8–15] and only a few series with small patient numbers have been reported to date [7, 16–19]. Recently, four large series of this rare disease have been reported [3–6]. However, all the data in these studies were obtained from public databases in the United States. As the patients were treated in various hospitals and the histological examination was conducted by various experts, misclassification bias might exist. Moreover, most of the patients enrolled in these studies did not undergo surgical resection, and detailed treatment information was not available for most of the patients, so specific treatment recommendations might not be drawn from these studies. Furthermore, the clinicopathological features of esophageal cancer, including the histology, tumor location, and age distribution, vary widely between patients in Eastern and Western countries. Lastly, the etiologic factors for esophageal cancer were also quite different between patients in Eastern and Western countries. The major risk factors for esophageal cancer in western countries were history of smoking, BMI above the lowest quartile, history of gastro-esophageal reflux, and low fruit and vegetable consumption [20]. However, history of smoking, alcohol consumption, drinking beverages at high temperatures, and poor nutritional status accounted for most of the esophageal cancer in eastern countries [20]. We think that more data on this rare disease from Eastern countries patients should be analyzed.

In the current study, we evaluated data from 56 patients with EASC who underwent esophagectomy from a single center and compared the clinicopathological features and prognosis of these patients with those of ESCC patients who underwent esophagectomy at the same time. All patients selected surgical resection as their initial treatment. All resection specimens were re-examined by an expert pathologist (Dr. Xiao-long Wei) to avoid misclassification bias. The homogeneity in histopathology and treatment may give us a more reliable understanding of this rare disease.

Due to the small volume of biopsy specimens from esophagoscopies, it is difficult to obtain an accurate pathological diagnosis before surgery [7, 16–19]. In this study, although 43 patients underwent esophagoscopic biopsy before treatment, only 1 patient (2.3%) was diagnosed with EASC, and all the other patients were misdiagnosed with ESCC. Ni et al. [17] reported that 92.1% (35/38) of patients were misdiagnosed with ESCC or others in preoperative esophagoscopic biopsy. Zhang et al. [16] reported that only 2 of the 18 patients (11.1%) were diagnosed with EASC in preoperative esophagoscopic biopsy, while 13 patients (72.2%) were misdiagnosed with ESCC and 3 patients (16.7%) were misdiagnosed with esophageal adenocarcinoma (EAC). One reason for the high rate of misdiagnosis may be that the squamous cell carcinoma component was mainly found in the epithelium, while the adenocarcinoma component mainly occurred in the submucosal gland or even deeper portion, which was difficult to be obtained from esophagoscopic biopsy [17]. So, most of these patients were misdiagnosed as ESCC in preoperative esophagoscopic biopsy. The other reason may be that the squamous and mucinous containing components are separate in adenosquamous carcinoma [1]. As the biopsy specimens from esophagoscopies were very small, it might be difficult to observe these two components simultaneously in these small specimens. The diagnosis of these carcinomas often requires resection specimens [2].

Data from the USA showed that the demographics and clinicopathological features of EASC were more similar to those of EAC than to those of ESCC [3–6]. For example, the male:female ratio was similar between EASC and EAC (approximately 6:1) but was significantly higher than that of ESCC (approximately 2:1) [3–6]. Nearly 70% of EASC was found in the lower third of the esophagus, which was similar to that of EAC but was significantly higher than the 30% for ESCC [3–6].. However, our data showed that the demographics and clinicopathological features of patients with EASC in China were different from those of patients with EASC in Western countries but were similar to those of patients with ESCC in China. Most of the EASC and ESCC cases were located in the middle third of the esophagus in this study, while only 17.9% of EASC and 16.5% of ESCC cases were located in the lower third of the esophagus (*P* = 0.737). Moreover, the male:female ratio was similar between EASC and ESCC (both approximately 3:1, *P* = 0.660). Furthermore, the mean age of patients with EASC at diagnosis in this study was 59.7 years, lower than that of approximately 66 years for patients with EASC in Western countries [3–6]. The differences in demographics and clinicopathological features of EASC between Eastern and Western countries patients may contribute to the different pathogenesis or tumor biology of this disease in different areas. We think that more data should be collected to investigate the potential differences in EASC between Eastern and Western countries patients.

Esophagectomy with lymphadenectomy is still the most important treatment for esophageal carcinoma, while neoadjuvant chemoradiotherapy is recommended for locally advanced disease [21]. Gamboa et al. [6] found that 20% of patients with EASC who received preoperative chemoradiotherapy had a pathologically complete response, which was similar to that of patients with EAC, and recommended that EASC should be treated more like EAC rather than ESCC. However, as it is difficult to obtain an accurate pathological diagnosis in preoperative esophagoscopic biopsy, most of these patients received surgical resection directly and received an accurate diagnosis from the resection specimens. It is reasonable to evaluate the value of postoperative adjuvant therapy in these patients. Our study showed that adjuvant chemotherapy was an independent predictor for patients with EASC after resection. Patients who received adjuvant chemotherapy had significantly better survival than patients who did not receive adjuvant chemotherapy. However, adjuvant radiotherapy did not improve survival for patients with EASC after resection.

Because of the rarity of EASC, the prognosis of this disease is still controversial. Most of the previous studies showed that EASC might be more aggressive than ESCC [4, 5, 7, 16]. However, Yendamuri et al. [3] reported that the survival was equivalent between patients with EASC and ESCC, but 32.7% of patients with EASC underwent surgical resection in their study, while only 15.9% of patients with ESCC underwent surgical resection. Yachida et al. [18] even found that EASC had a better prognosis than ESCC; however, half of the patients (9/18) in their cohort had T1 disease. Our previous study showed that the prognosis of EASC was similar to that of poorly differentiated ESCC but was significantly poorer than that of well- or moderately differentiated ESCC [7]. However, none of the previous studies matched the clinicopathological characteristics of patients with EASC and ESCC before survival analysis, which might contributed to these inconsistent results. In the current studies, we used PSM analysis to balance the baseline characteristics between patients with EASC and ESCC before survival analysis, and our results that the prognosis of EASC was similar to ESCC might be more reliable than previous studies.

Our study still has some limitations. First, it is a retrospective study from a single center, which may undermine its power. Second, the patient number in some subgroups was small, which limited its statistical power. Third, many other prognostic factors, such as the lymphovascular invasion, has been known to be a poor prognostic factor for many cancers. However, we did not recorded the information of these factors in this study. However, as there are few studies enrolling patients from Eastern countries concerned with this rare disease, our results may still provide us with a better understanding of this disease.

In conclusion, EASC is a rare disease and is easily misdiagnosed in esophagoscopic biopsy. The demographics and clinicopathological features may be different between patients with EASC from Eastern and Western countries. The prognosis of EASC was similar to that ESCC. Postoperative adjuvant chemotherapy may improve the survival of patients with EASC after esophagectomy. Further studies are needed to evaluate our findings and investigate a multidisciplinary treatment strategy for EASC.

## Data Availability

The datasets generated during and/or analyzed during the current study are not publicly available due to hospital regulations.

## Abbreviations

EASC: Esophageal adenosquamous carcinoma
ESCC: Esophageal squamous cell carcinoma
PSM: Propensity score matching
OS: Overall survival
MST: Median survival time
CI: Confidence interval
EAC: Esophageal adenocarcinoma
